# Agenesis of the Corpus Callosum with Facial Dysmorphism and Intellectual Disability in Sibs Associated with Compound Heterozygous *KDM5B* Variants

**DOI:** 10.3390/genes12091397

**Published:** 2021-09-10

**Authors:** Sébastien Lebon, Mathieu Quinodoz, Virginie G. Peter, Carole Gengler, Gaëlle Blanchard, Viviane Cina, Belinda Campos-Xavier, Carlo Rivolta, Andrea Superti-Furga

**Affiliations:** 1 Pediatric Neurology and Neurorehabilitation Unit, Woman Mother Child Department, Lausanne University Hospital (CHUV), 1011 Lausanne, Switzerland; 2 Medicine Department, Institute of Molecular and Clinical Ophthalmology Basel (IOB), 4056 Basel, Switzerland; mathieu.quinodoz@iob.ch (M.Q.); virginie.peter@iob.ch (V.G.P.); carlo.rivolta@iob.ch (C.R.); 3 Department of Ophthalmology, University of Basel, 4001 Basel, Switzerland; 4 Department of Genetics and Genome Biology, University of Leicester, Leicester LE1 7RH, UK; 5 Division of Pathology, Lausanne University Hospital (CHUV), 1011 Lausanne, Switzerland; carole.gengler@chuv.ch; 6 Paediatric Neurology Unit, Hopital de Fribourg, 1752 Fribourg, Switzerland; Gaelle.Blanchard@h-fr.ch; 7 Division of Genetic Medicine, Medicine Department, Lausanne University Hospital (CHUV), University of Lausanne, 1011 Lausanne, Switzerland; viviane.cina@chuv.ch (V.C.); Belinda.Campos-Xavier@chuv.ch (B.C.-X.); andrea.superti-furga@chuv.ch (A.S.-F.)

**Keywords:** corpus callosum agenesis, *KDM5B*

## Abstract

We studied a family in which the first-born child, a girl, had developmental delay, facial dysmorphism, and agenesis of the corpus callosum (ACC). The subsequent pregnancy was interrupted as the fetus was found to be also affected by ACC. Both cases were heterozygous for two *KDM5B* variants predicting *p* (Ala635Thr) and *p* (Ser1155Ala*fs*Ter4) that were shown to be in *trans*. *KDM5B* variants have been previously associated with moderate to severe developmental delay/intellectual disability (DD/ID), autism spectrum disorders (ASD), and dysmorphism in a few individuals, but the pathogenetic mechanisms are not clear yet as patients with both monoallelic and biallelic variants have been observed. Interestingly, one individual has previously been reported with ACC and severe ID in association with biallelic *KDM5B* variants. Together with the observations in this family, this suggests that agenesis of the corpus callosum may be part of the phenotypic spectrum associated with *KDM5B* variants and that the *KDM5B* gene should be included in gene panels to clarify the etiology of ACC both in the prenatal and postnatal setting.

## 1. Introduction

Agenesis of the corpus callosum (ACC) is one of the brain anomalies most frequently detected on prenatal imaging. Its pathogenesis is heterogeneous, as it can result from disruption of various pathophysiological mechanisms occurring at different steps of brain development (ranging from neuronal and glial proliferation, midline patterning, callosal neuron migration, and specification to guidance of commissural axons) [1]. ACC can be complete or partial; it is also prognostically relevant to distinguish whether it is isolated or part of a more complex malformation syndrome. The associated clinical manifestations form a wide spectrum ranging from fortuitously detected asymptomatic forms to severe neurodevelopmental disorders. In isolated ACC, intellectual disability is observed in approximatively 25% of cases only [2].

Because of this wide clinical spectrum, an extensive workup is necessary to provide accurate counseling in a prenatal setting. The diagnosis of “apparently isolated ACC”, implying a relatively benign prognosis, requires normal third-trimester fetal brain magnetic resonance imaging, absence of extra neurological malformations, consanguinity, or alcohol consumption, and normal array CGH analysis [3]. However, some forms of syndromic ACC associated with more or less marked facial dysmorphism and a poor developmental outcome are difficult to recognize in the prenatal ultrasound and can be easily misrecognized as “apparently isolated ACC”.

More than 1000 MIM (Mendelian Inheritance in Man) entities are associated with callosal abnormalities [1]. The development of Next Generation Sequencing technologies has led to the identification of many “novel” genetic causes of ACC. To date, 30–45% of callosal abnormalities are estimated to occur in a syndromic context and to have an identifiable genetic origin, most commonly single gene variants. In contrast, no single causative gene was identified for isolated ACC [4]. Here we report on the molecular workup of a family with two sibs affected by ACC that led us to identify compound heterozygous variants in *KDM5B* leading to a syndromic phenotype with facial dysmorphism and intellectual disability, adding *KDM5B* to the list of genes associated with callosal abnormalities.

## 2. Case Reports

**Figure 1:**
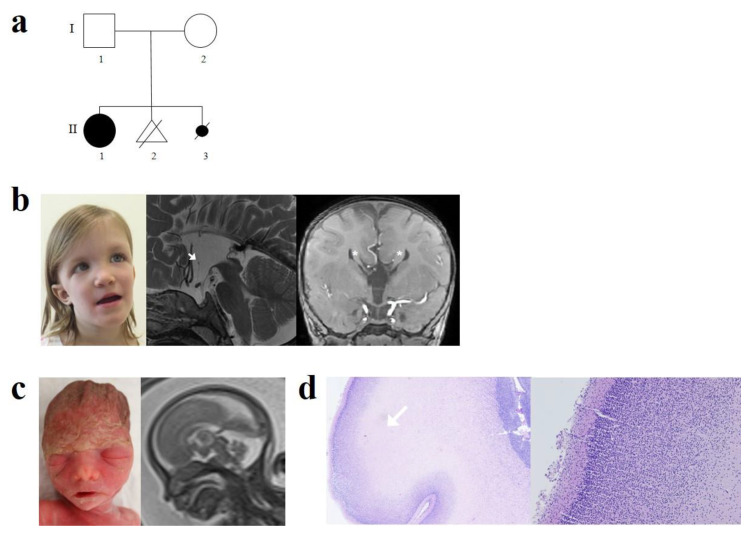
(**a**) Pedigree of the family. (**b**) II1: Photograph of the index case: note the large forehead, prominent metopic region, down slanting palpebral fissures, epicanthal fold, bulbous nasal tip and high nasal bridge, thin upper lip, low set ears. Brain MRI: Complete agenesis of the corpus callosum. Hypoplastic anterior commissure (white arrows), absence of interthalamic adhesion on T2-weighted sagittal sequence. Presence of Probst bundles (*) on T1-weighted coronal sequence. (**c**) II3: 24 GW fetus, mild hypertelorism. Fetal brain MRI at 22 GW: Truncated corpus callosum with absent splenium. (**d**) Histopathological examination of the fetal brain showing normal cortical laminar organization-hematoxylin-eosin, original magnification ×4 and ×10.

Case 1 was a full-term female, the first child of non-consanguineous parents of Swiss origin. Her father had a medical history of infantile epilepsy, not otherwise specified, and learning disorders. Pregnancy and delivery were uneventful. Apgar score was at 9/10/10 with normal birth weight (3360 g), length (51 cm), and head circumference (HC) (36 cm). Axial hypotonia and mild dysmorphic features consisting of saddle nose and low set ears were noted at day 4. Brain ultrasound, done in the neonatal period, was suggestive of ACC. At 3 months, she displayed poor visual contact and marked hypotonia. Fundoscopy showed bilateral optic hypoplasia. At this step, an endocrine workup was done given the suspicion of septo-optic dysplasia spectrum and was normal. She was able to sit at 11 months, crawl at 13 months, and walk at 25 months. Visual contact improved and was normal at 14 months. At four years, she had normal growth parameters (+0.5 SD for HC, 0 SD for weight, and +1 SD for height) and dysmorphic features consisting of large forehead, prominent metopic region, down slanting palpebral fissures, epicanthal fold, bulbous nasal tip and high nasal bridge, thin upper lip, low set ears, mild pectus excavatum, and umbilical hernia. She displayed severe developmental delay with poor speech (5 words) and hypotonia. Cerebral magnetic resonance imaging (MRI) showed complete agenesis of the corpus callosum with Probst bundles, hypoplastic anterior commissure, and absent interthalamic adhesion (Figure 1).

Case 2 was a fetus, sibling of case 1, from a subsequent pregnancy of this couple. Agenesis of the corpus callosum was suspected during the second-trimester ultrasound at 21 weeks of gestation (GW); fetal MRI at 22 GW confirmed a partial ACC with an absent splenium. No other cerebral abnormalities were noted. An array-CGH was normal. Given the family history of their first child with developmental delay and complete ACC, a poor developmental prognosis was predicted, and the couple chose to terminate the pregnancy at 24 GW. Fetal post-mortem examination showed a eutrophic female fetus with a head circumference of 22.5 cm (60th centile) and normal height and weight (50th centile for both). Mild hypertelorism but no other dysmorphic features were observed. Neuropathological findings revealed a brain that had normal weight (50th centile for age) and normal structure except for ACC on gross examination. Specifically, there were no cortical ribbon abnormalities or others brain micro-architecture anomalies.

## 3. Genetic Studies

Molecular studies were done in the older affected sib to identify the cause of her syndromic condition with facial dysmorphism, ACC, and intellectual disability. An array CGH (Illumina HumanOmniExpress chip, mean resolution of 300 kb) investigation was normal. A subsequent exome sequencing study (Illumina HiSeq2500 sequencer, Agilent Sure Select Human All Exon V7 capture kit, GrCh37) was done in 2018 with an analysis of approx. 1300 genes associated with a developmental disability did not yield diagnostic results. As the family was hoping for a molecular result for genetic counseling for reproductive planning, a reanalysis of the exome data was offered in 2019. This disclosed the presence of two heterozygous variants in the KDM5B gene: NM_001314042.1:c.1903G > A, *p* (Ala635Thr) as well as c.3463del, *p* (Ser1155AlafsTer4). Segregation studies in the family revealed that the affected fetus was compound heterozygous for the same variants and that the mother carried only the *p* (Ser1155AlafsTer4) variant. Although the father refused further studies, recurrence of the *p* (Ala635Thr) variant in the two sibs led us to consider that the variant must be paternal and thus in trans. Although we cannot formally exclude the possibility that the mother is fully heterozygous for one variant and mosaic for the second variant, this event has a very low likelihood. The missense variant was present at heterozygosity in a single individual from gnomAD, had a CADD score of 28.8, and was located inside a JmjC domain (UniProtKB-Q9UGL1). The alanine residue affected by the mutation is conserved in all vertebrates (multiz) and in human paralogues (KDM5A, KDM5C, and KDM5D). The frameshift variant was absent from the gnomAD database; it is located in exon 25 out of 29, which suggests that the aberrant mRNA molecule will likely be degraded by nonsense-mediated decay (NMD).

## 4. Discussion

Genetic variants in *KDM5B* have been previously associated with moderate to severe developmental delay/intellectual disability (DD/ID), autism spectrum disorders (ASD), and dysmorphism [5,6,7,8,9]. Here, we extend the phenotypic spectrum by reporting two siblings with agenesis of the corpus callosum (ACC).

Case 1 had a marked developmental delay, and her craniofacial dysmorphism was remarkably similar to that of previously reported cases. Except for a ventricular septal defect (VSD) in a single case [7], the individuals reported so far did not have an abnormal head circumference, major non-neurological malformations, or failure to thrive. This was also confirmed by our clinical observation of case 1 and by the normal extra neurological post-mortem findings in our fetal case (case 2).

To date, approximately a dozen cases with pathogenic *KDM5B* variants have been reported, all of whom had cognitive impairment ranging from mild to severe. Premature truncation variants as well as (more rarely) missense variants have been observed; the variants have been monoallelic in some cases and biallelic in others; missense variants are not clustering in a particular domain [5,6,7,8,9,10]. Thus, at this stage, the pathogenic mechanism (haploinsufficiency or recessive loss-of-function (LoF)) is not fully clear; indeed, both mechanisms may be valid. There is evidence that *KDM5B* haploinsufficiency may be associated with a phenotype of developmental delay, although its penetrance is incomplete [10]. The observation of a significant number of individuals with heterozygous stopgain or frameshift *KDM5B* variants in the gnomAD database (pLi of 0 and missense z-score of 1.78) suggests that LoFs variants are not necessarily always associated with an ID phenotype (incomplete penetrance). A recent report of a patient with a developmental disorder and a duplication involving four genes, including *KDM5B*, suggested that *KDM5B* hyperactivity might disrupt the expression of target genes and thus result in a neurodevelopmental disorder [11]. However, no experimental data support this hypothesis. Of note, the mother of our patients, who was a carrier of the frameshift/premature termination variant, had normal intelligence with no signs of developmental disability. Despite the absence of abnormal neurodevelopmental history in both parents, they did not have brain MRI to search for a familial form of ACC.

Given what was discussed above, there seem not to be easily recognizable phenotype-genotype correlations so far. However, one single case with ACC (and severe ID) and a homozygous LoF variant in *KDM5B* has been reported so far [7]; together with the two cases reported here, this might suggest the possibility of a more severe brain phenotype in the presence of biallelic variants.

Agenesis of the corpus callosum can result from the disruption of numerous developmental steps [1]. A brain MRI of the index case showed complete ACC with Probst bundles and hypoplastic anterior commissure suggesting a larger process of commissure development [12]. The absence of interthalamic adhesion is an aspecific finding, also found in the general population but overrepresented in midline malformations [13,14]. In our cases, it was more likely an incidental finding since it was not seen on the fetal MRI [14]. Neurohistopathological did not find any callosal structures, but Probst bundles, that are thick bundles of callosal axons that failed to cross the midline, were not identifiable. Of interest, the cortical ribbon had a normal six-layered organization, excluding major migration disorders.

*KDM5B* encodes an H3K4-specific eraser enzyme catalyzing the demethylation H3K4 [15] that plays an important role in early embryonic development, neuronal proliferation, and differentiation, two major steps for corpus callosum development [16]. Animal embryonic cellular models showed that *KDM5B* regulates cell fate decision to neuronal lineage, and knockdown embryonic stem cells results in failure to differentiate to neurons. Tight control of this demethylase is thus critical for cell fate determination. Poor neuronal specification might explain cognitive dysfunction, but also ACC, since the specification of neurons in the cortical plate is an essential process for callosal development [17]. In addition, transcription factors are known to act as an important regulator of callosal neuron specification [1,18]. As an example, *KMT2D* and *KMT2C* mutations are related to Kabuki and Kleefstra syndromes, respectively; two severe neurodevelopmental disorders associated with ID and corpus callosum hypoplasia [15].

In conclusion, we observed novel compound heterozygous *KDM5B* variants associated with impaired brain development, craniofacial dysmorphism, and ACC. This report does not allow formally excluding a possible coincidental association and would need to be validated by further reports. However, our data suggest that *KDM5B* may be a causative gene for syndromic ACC. In the prenatal setting (as seen in the second case in this family), the distinction between syndromic or isolated ACC may be difficult or impossible as syndromic features may not easily be recognized. Based on these observations, it seems reasonable to include *KDM5B* in diagnostic gene panels for ACC.

## Data Availability

No new data were created or analyzed in this study. Data sharing is not applicable to this article.
